# Supplementary material for: Structure and mechanism of cancer-associated N-acetylglucosaminyltransferase-V
Source: Nat Commun. 2018 Aug 23;9:3380. doi: 10.1038/s41467-018-05931-w (PMC6107550; doi:10.1038/s41467-018-05931-w)
Supplement: Supplementary file 1 — Supplementary Information [file 41467_2018_5931_MOESM1_ESM.pdf]

Supplementary Information

Structure and mechanism of cancer-associated N-acetylglucosaminyltransferase-V

Nagae et al.

Supplementary Table 1 Nucleotide sequences of the primers used in this study

A restriction enzyme site (XhoI) is underlined.

Primer	Nucleotide sequence
pcDNA-IH/GnT-V T121 fwd	5’-CTACAGCTGTTCCCAGCTTG-3’
pcDNA-IH/GnT-V L741 rev	5’-AGGCTCGAGCTATAGGCAGTCTTTGCAGA-3’
N_del_fwd	5’- AGATCCGTCCTTTCCCTGGAAGTAC-3’
N_del_rev	5’- AATTCATTGGCGGAAATTCGTACAG-3’
Loop_change_fwd	5’-CTTCATGATTTCCTTGAGCTCAGCC-3’
Loop_change_rev	5’-ggcggcggcggcATTGTTGAGCTCATTTACATTGATA-3’
E280A (fwd and rev)	5’-CCTGGGACTCCTGACCAAGGCATCTGGATTTAAGATTGCAG-3’<br>5’-CTGCAATCTTAAATCCAGATGCCTTGGTCAGGAGTCCCAGG-3’
E287A (fwd and rev)	5’-ATCTGGATTTAAGATTGCAGCGACAGCTTTCAGTGGTGGCC-3’<br>5’-GGCCACCACTGAAAGCTGTCGCTGCAATCTTAAATCCAGAT-3’
E297A (fwd and rev)	5’-CAGTGGTGGCCCTCTTGGTGCATTAGTTCAATGGAGTGATT-3’<br>5’-AATCACTCCATTGAACTAATGCACCAAGAGGGCCACCACTG-3’
E429A (fwd and rev)	5’-CTTTCTGGGGTTTGTGGTTGCGCAGCACCTGAACTCCAGTG-3’<br>5’-CACTGGAGTTCAGGTGCTGCGCAACCACAAACCCCAGAAAG-3’
E520A (fwd and rev)	5’-TGGACTTGGGTTCCCTTACGCGGGCCCAGCTCCCCTGGAAG-3’<br>5’-CTTCCAGGGGAGCTGGGCCCGCGTAAGGGAACCCAAGTCCA-3’
E526A (fwd and rev)	5’-CGAGGGCCCAGCTCCCCTGGCAGCTATCGCAAATGGATGTG-3’<br>5’-CACATCCATTTGCGATAGCTGCCAGGGGAGCTGGGCCCTCG-3’

Supplementary Table 2

Interaction network between acceptor trisaccharide and mini-GnT-V

(i) Direct protein-glycan and (ii) water-mediated interactions of nitrogen and oxygen atoms whose distances are within 3.5 Å. The water-mediated interactions listed below are common in the two complexes in the asymmetric unit.

(i) Direct interaction

Trisaccharide	Distance (Complex A, B)	GnT-V
Man-2 O3	2.6, 2.9	K554 Nζ
Man-2 O3	3.4, -	K554 Nζ
GlcNAc-3 O3	2.9, 3.2	S379 N
GlcNAc-3 O3	3.5, 3.5	S379 Oγ
GlcNAc-3 O4	2.5, 2.5	D378 Oδ
GlcNAc-3 O5	2.8, 3.0	K554 Nζ
GlcNAc-3 O6	3.3, 3.0	K554 Nζ
GlcNAc-3 O7	3.1, -	S379 Oγ

(ii) Water-mediated indirect interaction

<table>
<tr><th>Trisaccharide</th><th>Distance (A, B)</th><th>Water (A, B)</th><th>Distance (A, B)</th><th>GnT-V</th></tr>
<tr><td>GlcNAc-3 O3</td><td>2.8, 2.6</td><td>Wat69, 190</td><td>3.0, 2.8</td><td>L377 O</td></tr>
<tr><td>GlcNAc-3 O3</td><td>3.2, 3.3</td><td rowspan="2">Wat154, 52</td><td>3.0, 3.0</td><td>I353 N</td></tr>
<tr><td>GlcNAc-3 O4</td><td>2.8, 2.9</td><td>2.6, 2.7</td><td>L377 O</td></tr>
<tr><td rowspan="4">GlcNAc-3 O6</td><td rowspan="4">2.6, 2.6</td><td rowspan="4">Wat28, 172</td><td>2.5, 2.5</td><td>D378 Oδ1</td></tr>
<tr><td>3.5, 3.4</td><td>D378 Oδ2</td></tr>
<tr><td>3.2, 3.4</td><td>G381 N</td></tr>
<tr><td>2.7, 2.9</td><td>G381 O</td></tr>
</table>

Supplementary Table 3

Gel filtration analysis of GnT-V luminal domain and mini-GnT-V E297A proteins.

The gel filtration column, Superdex75 HiLoad16/60 (GE Healthcare), was used for this experiment. The molecular weights and elution points of standard proteins were as follows: Alcohol dehydrogenase (141kDa, 52.2mL), Bovine Serum Albumin (66.5kDa, 57.0mL) and Carbonic Anhydrase (29kDa, 72.4mL).

Proteins	Calculated MW (kDa)	Elution points (mL)	Estimated MW (kDa)
GnT-V luminal domain	71	57.9	81
Mini-GnT-V E297A	59	63.0	53.8

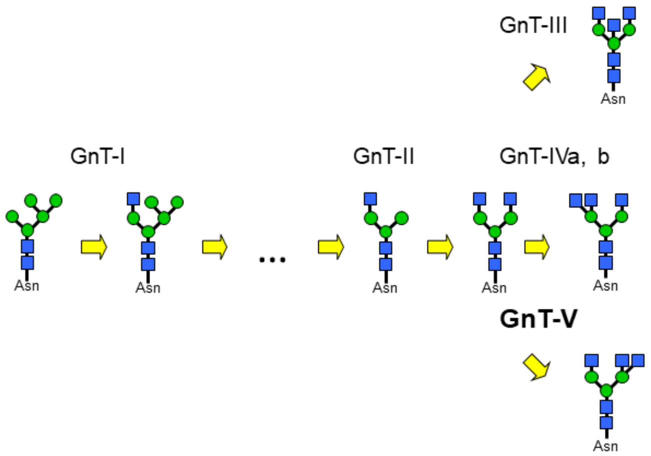

	GnT-I	GnT-II	GnT-III	GnT-IVa, b	**GnT-V**
CAZy databese	GT13	GT16	GT17	GT54	**GT18**
Fold	GT-A	GT-A	Unknown	Unknown	**GT-B**
Divalent ion	+	+	+	+	**-**
UDP-GlcNAc K_m (mM)	0.04	0.96	0.42	5.0	**11.0**
Acceptor K_m (mM)	2.0	0.19	1.2	0.73	**0.087**

Supplementary Figure 1

Biosynthetic pathway of GlcNAc-branched N-glycans catalyzed by human GlcNAc transferases (GnT-I~V) is shown in upper panel. Enzymatic properties of human GlcNAc transferases are summarized in lower Table. This figure was modified from original reviews[1,2] and recent report about crystallographic study of GnT-II[3].

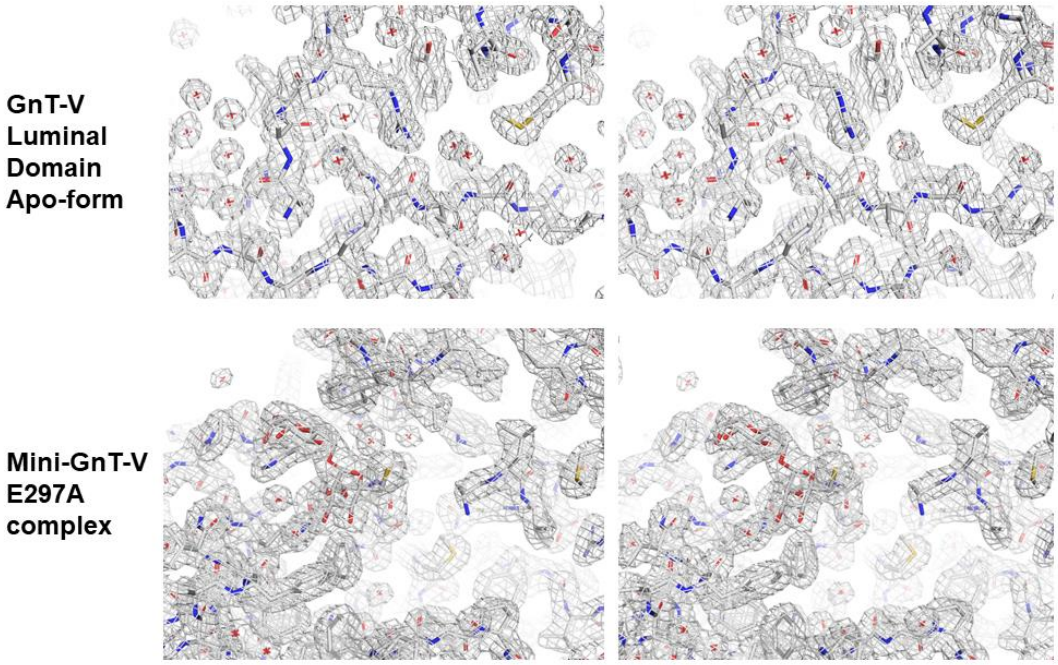

Supplementary Figure 2

The electron density map of refined structures. Stereo views of $2F_{obs}$-F_{calc} electron density map contoured at 1.0 σ level of GnT-V luminal domain (upper panel) and Mini-GnT-V E297A inhibitor complex (lower panel) are shown.

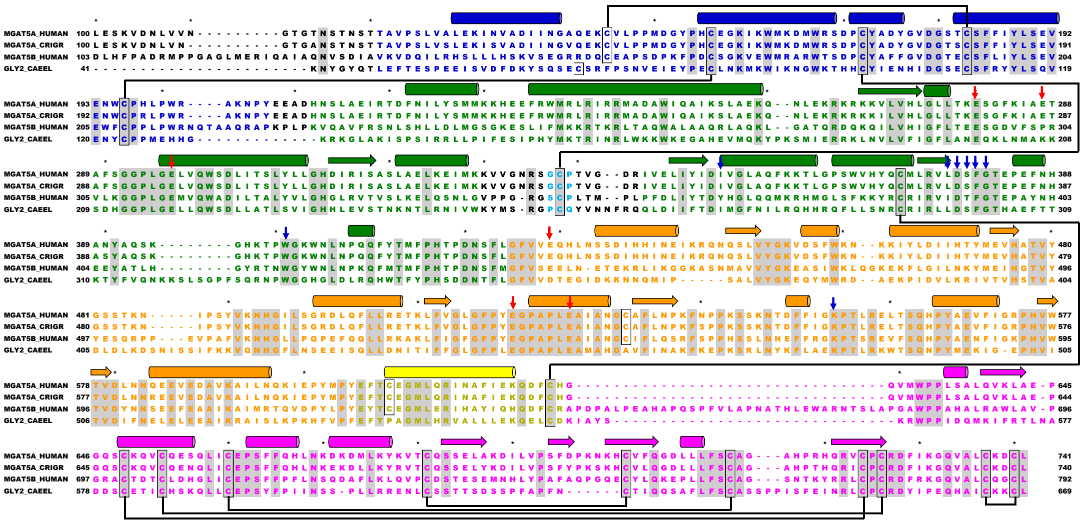

Supplementary Figure 3

Amino acid sequence alignment of GnT-V and related GT18 family members. The alignment was performed with ClustalW server. Accession database numbers of each sequence are as follows: Q09328 (human GnT-V, MGAT5A_HUMAN), G3GZF3 (Chinese Hamster GnT-V, MGAT5A_CRIGR), Q3V5L5 (human GnT-IX, MGAT5B_HUMAN), and Q9NDH7 (*C.elegans* Gly-2, GLY2_CAEEL). Cysteine residues are enclosed in boxes and disulfide bond pattern is indicated. Secondary structural elements of human GnT-V are indicated above the sequence. Six glutamates subjected to mutational experiments, are indicated with red arrows. Amino acid residues which interact with acceptor trisaccharide are indicated with blue arrows. The amino acid sequence identities with human GnT-V are 97% (Chinese Hamster GnT-V), 40% (human GnT-IX) and 28% (*C.elegans* Gly-2).

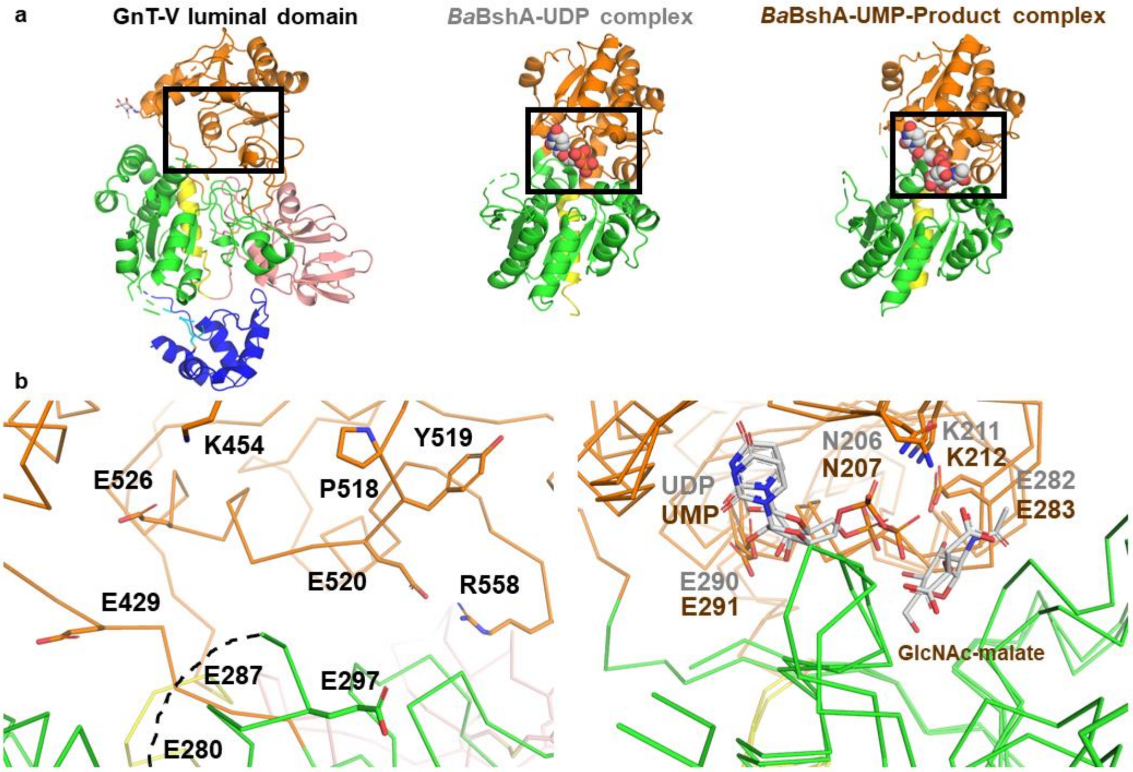

Supplementary Figure 4

Structural comparison between GnT-V luminal domain and bacterial glycosyltransferase, *Ba*BshA

(a) Structural comparison between GnT-V luminal domain (left panel), bacterial glycosyltransferase, *Ba*BshA-UDP complex (middle panel, PDB code: 3MBO[4]) and BaBshA-UMP-GlcNAc-malate complex (right panel, PDB code: 5D00[5]). Protein and ligand molecules are shown in ribbon and sphere models, respectively. Three structures are depicted from the same view angles. Ligand binding sites are highlighted in black boxes.

(b) Structural comparison of donor and product binding sites of *Ba*BshA (right panel) and corresponding region of GnT-V luminal domain (left panel). Two structures of BaBshA complexes are superimposed in right panel. Polar residues which interact with UDP in *Ba*BshA and corresponding residues in GnT-V are shown in rod models. Also, six glutamates applied to mutational experiments are shown in rod models.

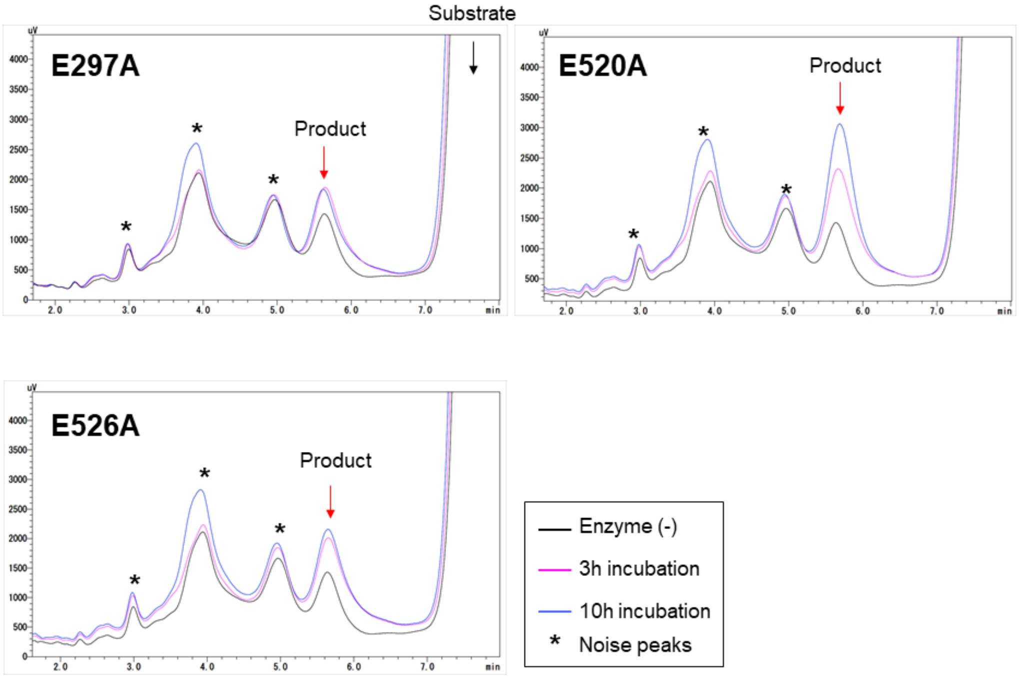

Supplementary Figure 5

Enzymatic activity of three GnT-V mutants (E297A, E520A and E526A) with prolonged reaction time.

Elution profiles of the substrate and product after the incubation of each mutant with the substrate for three (pink) or ten (blue) hours. The positions of the product were indicated.

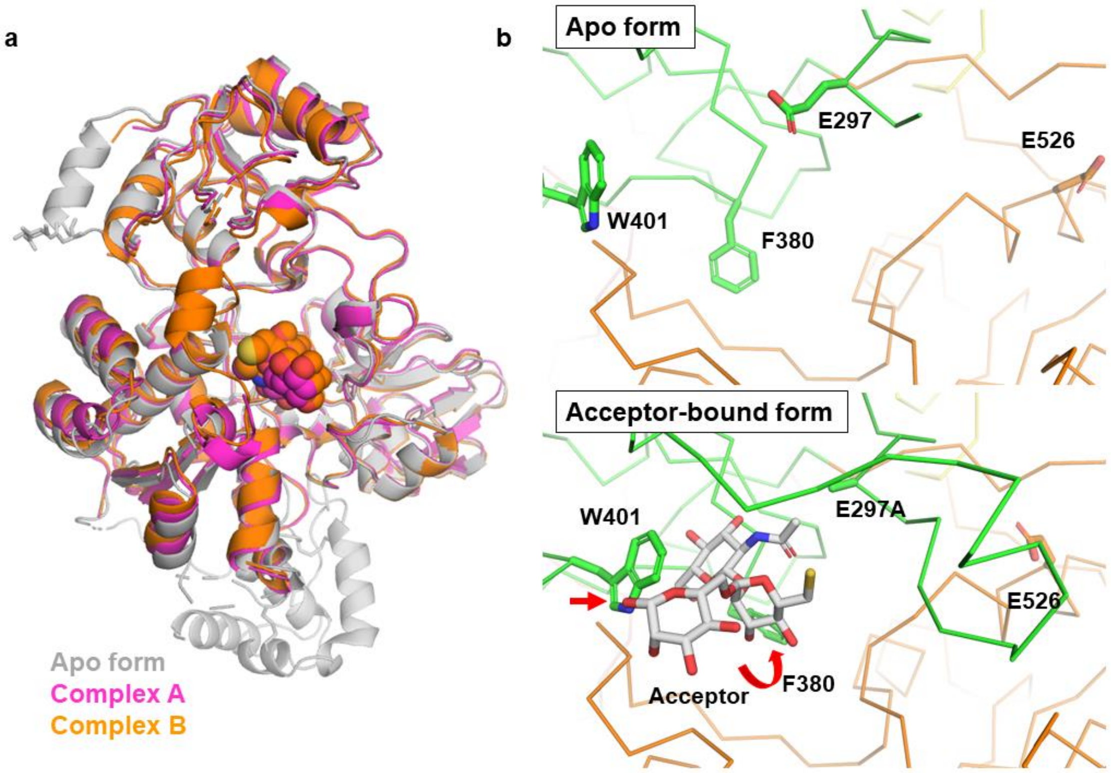

Supplementary Figure 6

(a) Structural superposition of apo form (grey) and two complexes (A: magenta, B: orange) in the asymmetric unit. Proteins and ligands are shown in ribbon and sphere models, respectively.

(b) Structural comparison between apo form (upper panel) and acceptor-bound form (lower panel) focusing on the catalytic center. Sugar residues and amino acid residues involved in catalytic reaction are shown in rod models and labeled. Observed structural differences upon acceptor binding are indicated with red arrows.

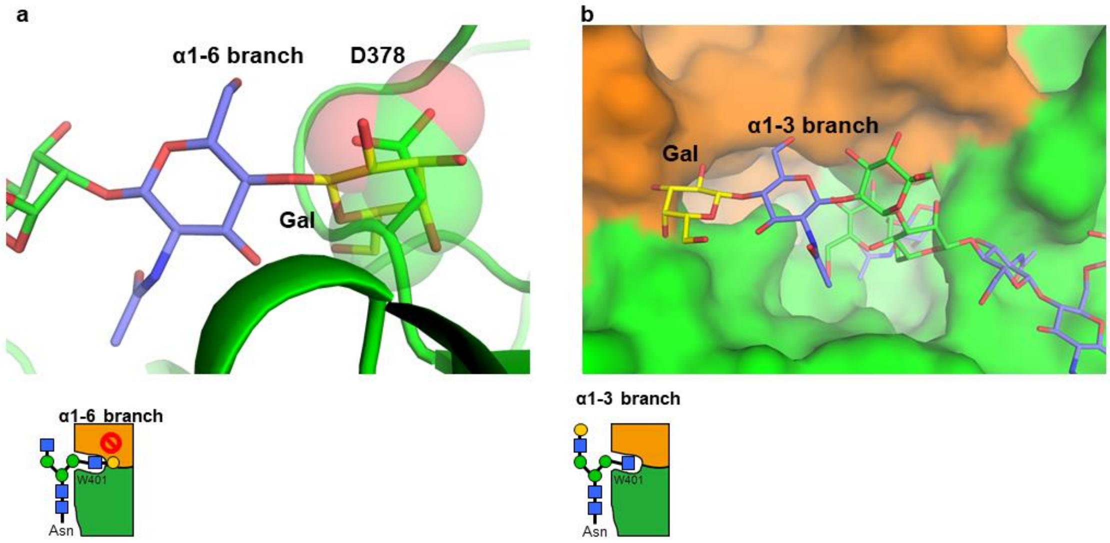

Supplementary Figure 7

(a) Docking models of α1-3 branch of biantennary glycan onto α1-6 branch. As described in main text, β-mannose causes steric clash with W401. In addition, chitobiose also causes heavy clash with S281-G282.

(b) Docking models of galactosylated N-glycans with GnT-V

Left panel: Galactose extension at α1-6 branch. Structural superposition of galactosylated α1-6 branch onto the crystal structure of mini-GnT-V trisaccharide complex. The galactose sterically clashes with D378 of mini-GnT-V.

Right panel: Galactose extension at α1-3 branch. N-glycan is shown in rod model. Mini-GnT-V is shown in surface model.

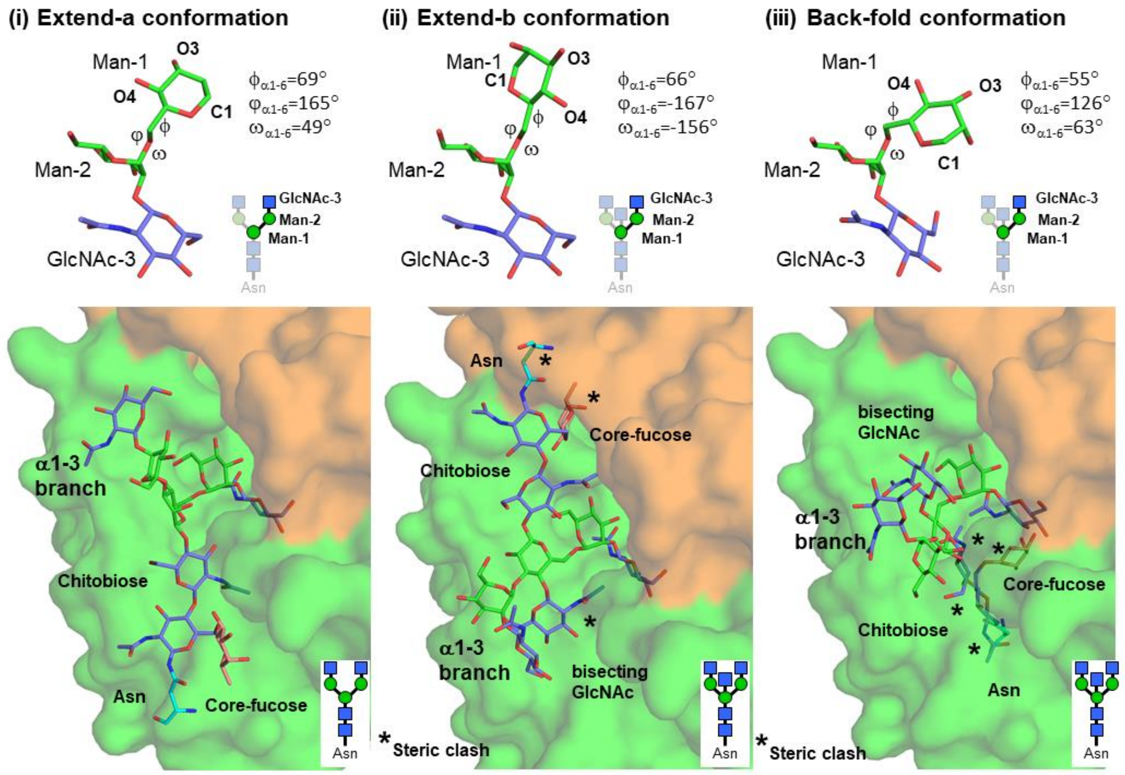

Supplementary Figure 8

Conformation selective inhibition mechanism of GnT-V. The dihedral angles ($\phi_{\alpha1\text{-}6}$, $\varphi_{\alpha1\text{-}6}$, $\omega_{\alpha1\text{-}6}$) of α1-6 linkages between Man-1 (β-mannose) and Man-2 affect global conformation of N-glycans. Three different conformations of trisaccharide unit (GlcNAcβ1-2Manα1-6Man) are shown in upper panels and docking models of three whole N-glycans onto mini-GnT-V are shown in lower panels.

(i) Trisaccharide unit of extend-a conformation ($\phi_{\alpha1\text{-}6}$=69°, $\varphi_{\alpha1\text{-}6}$=165°, $\omega_{\alpha1\text{-}6}$=49°) of biantennary N-glycan observed in crystal structure is shown in upper panel. Docking model of full N-glycan fits well into the cavity of mini-GnT-V is shown in lower panel.

(ii) Trisaccharide unit of extend-b conformation ($\phi_{\alpha1\text{-}6}$=66°, $\varphi_{\alpha1\text{-}6}$=-167°, $\omega_{\alpha1\text{-}6}$=-156°) of bisected N-glycan retrieved from the crystal structure of bisected N-glycan in complex with murine DCIR2 (PDB code: 3VYK, [6]) is shown in upper panel. Docking model of bisected glycan complex is shown in lower panel. Compared with extend-a conformation, relative orientation of stem asparagine is flipped. It causes steric clash with mini-GnT-V. Steric clashes with mini-GnT-V are indicated with asterisks.

(iii) Trisaccharide unit of back-fold conformation ($\phi_{\alpha1\text{-}6}$=55°, $\varphi_{\alpha1\text{-}6}$=126°, $\omega_{\alpha1\text{-}6}$=63°) of bisected N-glycan extracted from the crystal structure of bisected N-glycan in complex with PHA-E (PDB code: 5AV7, [7]) is shown in upper panel. Docking model with mini-GnT-V is displayed in lower

panel. Compared with extend-a conformation, chitobiose and core asparagine residues are deeply buried inside mini-GnT-V. Steric clashes are also indicated with black asterisks.

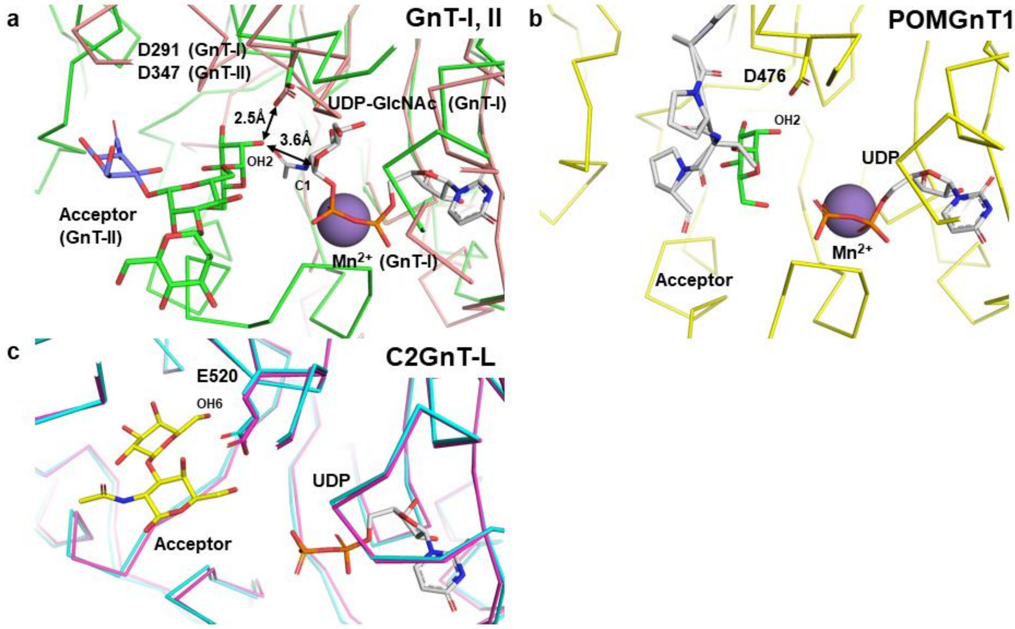

Supplementary Figure 9

Close-up views of substrate binding sites of other mammalian GlcNAc transferases which have GT-A folds.

(a) Close-up view of GnT-II-acceptor complex (PDB code: 5VCS, [3]) posed onto GnT-I-Mn^{2+}-UDP-GlcNAc complex (PDB code: 1FOA, [8]). The catalytic base residues are located at same positions. The distance between OH2 of acceptor in GnT-II and D347 is 2.5Å, whereas the distance between OH2 of acceptor in GnT-II and C1 of UDP-GlcNAc in GnT-I is 3.6Å.

(b) Close-up view of POMGnT1 in complex with UDP, Mn^{2+} ion and acceptor Glycopeptide (PDB code: 5GGI, [9])

(c) Close-up view of C2GnT-L in complex with acceptor disaccharide (PDB code: 2GAM, [10]) posed onto UDP complex (PDB code: 3OTK, [11]).

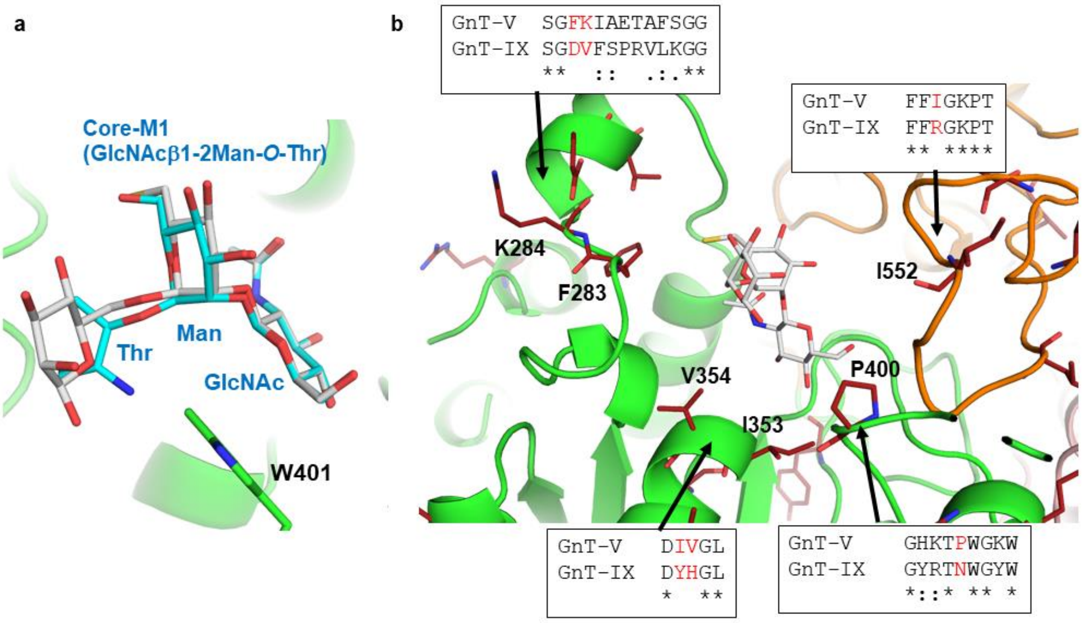

Supplementary Figure 10

Structure-based sequence comparison with GnT-IX (GnT-Vb)

(a) Hypothetical structure of Core-M1 glycan (GlcNAcβ1-2Man-*O*-Thr, cyan) was superposed onto mini-GnT-V trisaccharide (GlcNAcβ1-2Manα1-6Man) complex. Sugar residues and W401 are shown in rod model.

(b) Structure-based sequence comparison between GnT-V and IX. Incompatible amino acid residues are shown in rod model. Seven amino acid residues which are different in GnT-IX are shown in rod models and labeled. Partial sequence alignments around acceptor binding site are indicated with boxes.

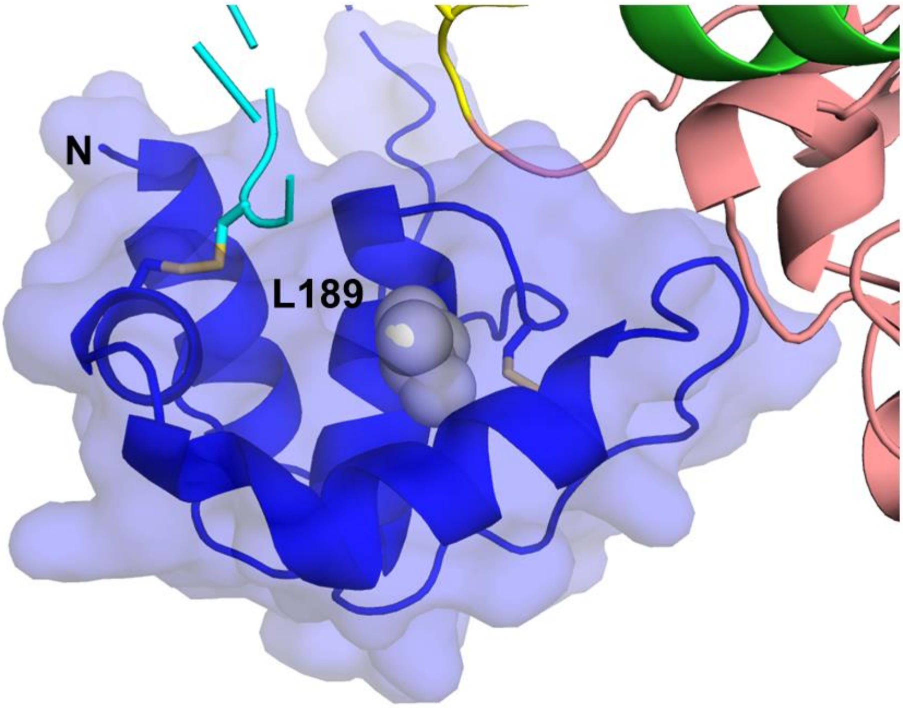

Supplementary Figure 11

Close-up view of N-terminal domain. N-terminal domain is shown in ribbon and semi-transparent surface models. The replaced residue in Lec4A mutant (L189R) is highlighted in sphere model.

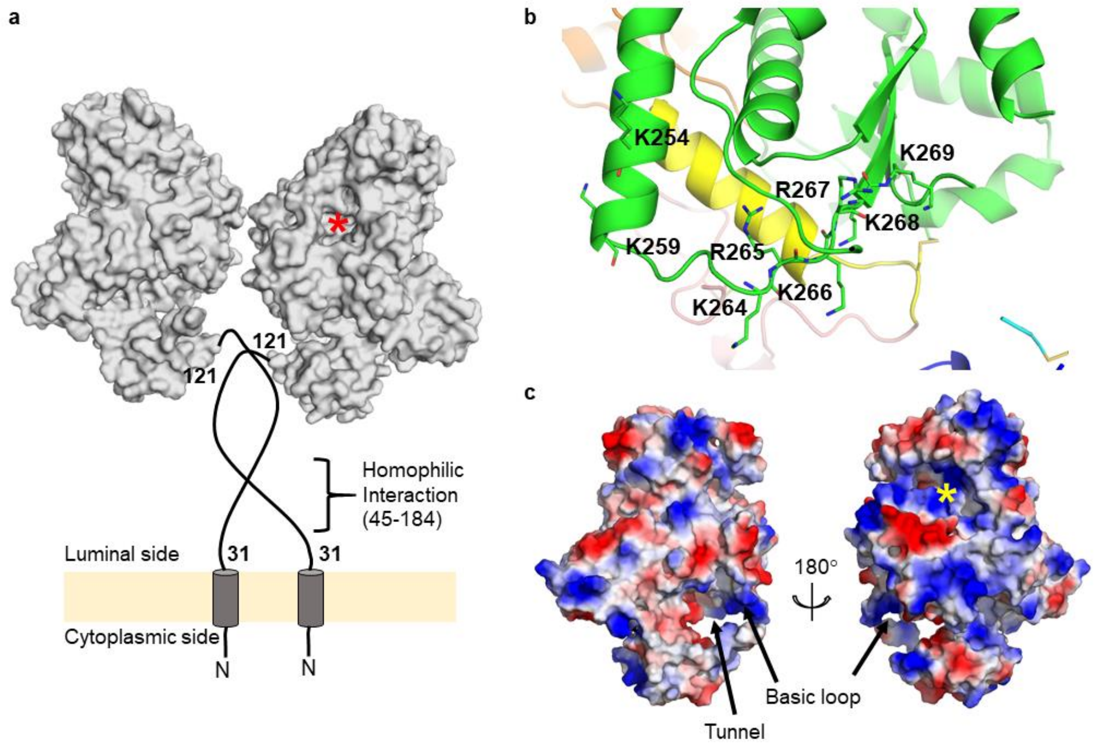

Supplementary Figure 12

(a) Hypothetical dimer formation via homophilic interaction region. The catalytic center is indicated with red asterisk.

(b) Close-up view of positively charged loop (K264-K269) and adjacent lysine residues (K254 and K259) in middle domain 1. Positively charged residues are shown in rod models.

(c) Electrostatic surface representation of GnT-V luminal domain. The position of catalytic center is indicated with asterisk. The position of basic loop and adjacent tunnel are also labeled.

Supplementary References

1. Varki, A. *Essentials of glycobiology*, xxix, 823 pages (Cold Spring Harbor Laboratory Press, Cold Spring Harbor, New York, 2017).
2. Dennis, J.W., Nabi, I.R. & Demetriou, M. Metabolism, cell surface organization, and disease. *Cell* **139**, 1229-41 (2009).
3. Kadirvelraj, R. et al. Human N-acetylglucosaminyltransferase II substrate recognition uses a modular architecture that includes a convergent exosite. *Proc Natl Acad Sci U S A* **115**, 4637-4642 (2018).
4. Parsonage, D. et al. Characterization of the N-acetyl-alpha-D-glucosaminyl l-malate synthase and deacetylase functions for bacillithiol biosynthesis in Bacillus anthracis. *Biochemistry* **49**, 8398-414 (2010).
5. Winchell, K.R. et al. A Structural, Functional, and Computational Analysis of BshA, the First Enzyme in the Bacillithiol Biosynthesis Pathway. *Biochemistry* **55**, 4654-65 (2016).
6. Nagae, M. et al. Recognition of bisecting N-acetylglucosamine: structural basis for asymmetric interaction with the mouse lectin dendritic cell inhibitory receptor 2. *J Biol Chem* **288**, 33598-610 (2013).
7. Nagae, M. et al. Atomic visualization of a flipped-back conformation of bisected glycans bound to specific lectins. *Sci Rep* **6**, 22973 (2016).
8. Unligil, U.M. et al. X-ray crystal structure of rabbit N-acetylglucosaminyltransferase I: catalytic mechanism and a new protein superfamily. *EMBO J* **19**, 5269-80 (2000).
9. Kuwabara, N. et al. Carbohydrate-binding domain of the POMGnT1 stem region modulates O-mannosylation sites of alpha-dystroglycan. *Proc Natl Acad Sci U S A* **113**, 9280-5 (2016).
10. Pak, J.E. et al. X-ray crystal structure of leukocyte type core 2 beta1,6-N-acetylglucosaminyltransferase. Evidence for a convergence of metal ion-independent glycosyltransferase mechanism. *J Biol Chem* **281**, 26693-701 (2006).
11. Pak, J.E., Satkunarajah, M., Seetharaman, J. & Rini, J.M. Structural and mechanistic characterization of leukocyte-type core 2 beta1,6-N-acetylglucosaminyltransferase: a metal-ion-independent GT-A glycosyltransferase. *J Mol Biol* **414**, 798-811 (2011).
